# Changes in Suicide Rates — United States, 2018–2019

**DOI:** 10.15585/mmwr.mm7008a1

**Published:** 2021-02-26

**Authors:** Deborah M. Stone, Christopher M. Jones, Karin A. Mack

**Affiliations:** ^1^Division of Injury Prevention, National Center for Injury Prevention and Control, CDC; ^2^Office of the Director, National Center for Injury Prevention and Control, CDC.

Suicide is the 10th leading cause of death in the United States overall, and the second and fourth leading cause among persons aged 10–34 and 35–44 years, respectively (*1*). In just over 2 decades (1999–2019), approximately 800,000 deaths were attributed to suicide, with a 33% increase in the suicide rate over the period (*1*). In 2019, a total of 12 million adults reported serious thoughts of suicide during the past year, 3.5 million planned a suicide, and 1.4 million attempted suicide (*2*). Suicides and suicide attempts in 2019 led to a lifetime combined medical and work-loss cost (i.e., the costs that accrue from the time of the injury through the course of a person’s expected lifetime) of approximately $70 billion (https://wisqars.cdc.gov:8443/costT/). From 2018 to 2019, the overall suicide rate declined for the first time in over a decade (*1*). To understand how the decline varied among different subpopulations by demographic and other characteristics, CDC analyzed changes in counts and age-adjusted suicide rates from 2018 to 2019 by demographic characteristics, county urbanicity, mechanism of injury, and state. Z-tests and 95% confidence intervals were used to assess statistical significance. Suicide rates declined by 2.1% overall, by 3.2% among females, and by 1.8% among males. Significant declines occurred, overall, in five states. Other significant declines were noted among subgroups defined by race/ethnicity, age, urbanicity, and suicide mechanism. These declines, although encouraging, were not uniform, and several states experienced significant rate increases. A comprehensive approach to prevention that uses data to drive decision-making, implements prevention strategies from CDC’s Preventing Suicide: A Technical Package of Policy, Programs, and Practices with the best available evidence, and targets the multiple risk factors associated with suicide, especially in populations disproportionately affected, is needed to build on initial progress from 2018 to 2019 (*3*).

Data from the 2018–2019 National Vital Statistics System multiple cause-of-death mortality files were analyzed. Suicide deaths were identified by using *International Classification of Diseases, Tenth Revision* underlying cause-of-death codes U03, X60–X84, and Y87.0. Two-digit age-adjusted death rates (per 100,000 population) and confidence intervals were calculated by using the direct method and the 2000 U.S. standard population, rounded to one digit. Data for persons aged <10 years are not shown in results by age group because determining suicidal intent in younger children is difficult and case counts were <20, indicating unstable rates (*4*). Urbanization level of the decedent’s county of residence was categorized by using the 2013 National Center for Health Statistics Urban–Rural Classification Scheme for Counties (https://www.cdc.gov/nchs/data_access/urban_rural.htm). The classification levels for counties are as follows: 1) large central metropolitan: part of a metropolitan statistical area with ≥1 million population and covers a principal city; 2) large fringe metropolitan: part of a metropolitan statistical area with ≥1 million population but does not cover a principal city; 3) medium metropolitan: part of a metropolitan statistical area with ≥250,000 but <1 million population; 4) small metropolitan: part of a metropolitan statistical area with <250,000 population; 5) micropolitan (nonmetropolitan): part of a micropolitan statistical area (has an urban cluster of ≥10,000 but <50,000 population); and 6) noncore (nonmetropolitan): not part of a metropolitan or micropolitan statistical area.

Changes in suicide rates from 2018 to 2019 were examined overall and by age, sex, race/ethnicity, county urbanization level, mechanism of injury, and state. Single-race estimates are presented and might not be comparable to earlier years produced by bridging multiple race to a single race choice (https://wonder.cdc.gov/wonder/help/mcd-expanded.html). Hispanic and unknown ethnicity include persons of any race. Racial groups exclude persons of Hispanic or unknown ethnicity. Differences in rates between 2018 and 2019 were assessed by using z-tests when deaths were ≥100 and by using nonoverlapping confidence intervals based on a gamma distribution when deaths were <100; p-values <0.05 were considered statistically significant (*5*). Relative and absolute changes in rates were calculated; however, only relative changes are presented in the text.

In 2019, a total of 47,511 deaths were attributable to suicide. From 2018 to 2019, the overall suicide rate declined significantly by 2.1% (14.2 per 100,000 population to 13.9) (Table); among females, the rate declined by 3.2% (6.2 to 6.0) and among males by 1.8% (22.8 to 22.4). Among racial/ethnic groups, rates of suicide were highest in 2019 among American Indian/Alaskan Native (AI/AN) persons (22.5 per 100,000) overall, and among AI/AN females and males. Counts of suicide were highest among White persons (37,428). White persons were the only race for whom rates significantly declined from 2018 to 2019, declining 2.2% (18.1 to 17.7) overall, and declining significantly among females and males. Suicide rates did not significantly change from 2018 to 2019 for any other racial/ethnic group examined.

**TABLE:** Annual number and age-adjusted* rate of suicide^†^ per 100,000 population, by selected characteristics — National Vital Statistics System, United States, 2018 and 2019

Characteristic	2018	2019	Absolute change^§^	Relative change^¶^
	No. (rate) [95% CI]	No. (rate) [95% CI]		
**Overall**				
**Total**	**48,344 (14.2) [14.1–14.4]**	**47,511 (13.9) [13.8–14.1]**	–0.3**	–2.1**
**Race/Ethnicity^††^**				
American Indian/Alaska Native	545 (22.3) [20.4–24.2]	546 (22.5) [20.5–24.4]	0.2	0.9
Asian	1,315 (6.7) [6.4–7.1]	1,342 (6.7) [6.3–7.1]	0	0
Black or African American	3,022 (7.3) [7.0–7.5]	3,115 (7.5) [7.2–7.8]	0.2	2.7
Native Hawaiian/Other Pacific Islander	73 (11.9) [9.3–15.0]	90 (14.4) [11.5–17.7]	2.5	21.0
White	38,415 (18.1) [17.9–18.3]	37,428 (17.7) [17.5–17.9]	–0.4**	–2.2**
Multiracial	514 (9.0) [8.1–9.8]	527 (8.8) [8.0–9.6]	–0.2	–2.2
Hispanic	4,313 (7.4) [7.2–7.7]	4,331 (7.3) [7.0–7.5]	–0.1	–1.4
Unknown	147 (—)	132 (—)	—	—
**Age group, yrs^§§^**				
10–14	596 (2.9) [2.6–3.1]	534 (2.6) [2.4–2.8]	–0.3	–10.3
15–24	6,211 (14.5) [14.1–14.8]	5,954 (14.0) [13.6–14.3]	–0.5**	–3.4**
25–34	8,020 (17.6) [17.2–17.9]	8,059 (17.5) [17.2–17.9]	0.1	–0.6
35–44	7,521 (18.2) [17.8–18.6]	7,525 (18.1) [17.7–18.5]	–0.1	–0.5
45–54	8,345 (20.0) [19.6–20.5]	8,012 (19.6) [19.2–20.0]	–0.4	–2.0
55–64	8,540 (20.2) [19.8–20.6]	8,238 (19.4) [19.0–19.8]	–0.8**	–4.0**
65–74	4,974 (16.3) [15.9–16.8]	4,867 (15.5) [15.0–15.9]	–0.8**	–4.9**
75–84	2,880 (18.7) [18.0–19.4]	2,977 (18.6) [18.0–19.3]	–0.1	–0.5
≥85	1,248 (19.1) [18.0–20.1]	1,329 (20.1) [19.0–21.2]	1.0	5.2
**Urbanization^¶¶^**				
Large central metropolitan	11,978 (11.4) [11.2–11.6]	11,762 (11.2) [11.0–11.4]	–0.2	–1.8
Large fringe metropolitan	11,028 (13.0) [12.7–13.2]	10,840 (12.6) [12.4–12.8]	–0.4**	–3.1**
Medium metropolitan	10,862 (15.4) [15.1–15.7]	10,789 (15.2) [14.9–15.5]	–0.2	–1.3
Small metropolitan	5,373 (17.6) [17.1–18.0]	5,327 (17.4) [16.9–17.9]	–0.2	–1.1
Micropolitan (nonmetropolitan)	5,337 (19.2) [18.6–19.7]	5,009 (18.1) [17.6–18.6]	–1.1**	–5.7**
Noncore (nonmetropolitan)	3,766 (19.7) [19.0–20.4]	3,784 (20.1) [19.5–20.8]	0.4	2.0
**Mechanism of injury**				
Cut/Pierce	897 (0.3) [0.3–.03]	921 (0.3) [0.2–0.3]	0	0
Drowning	522 (0.1) [0.1–0.2]	506 (0.2) [0.1–0.2]	0.1	100***
Fall	1,149 (0.4) [0.3–0.4]	1,183 (0.4) [0.3–0.4]	0	0
Fire/Flame	214 (0.1) [0.1–0.1]	187 (0.1) [0–0.1]	0	0
Firearm	24,432 (7.0) [7.0–7.1]	23,941 (6.8) [6.8–6.9]	–0.2**	–2.9**
Poisoning	6,237 (1.8) [1.7–1.8]	6,125 (1.8) [1.7–1.8]	0	0
Suffocation	13,840 (4.3) [4.2–4.4]	13,563 (4.2) [4.1–4.3]	–0.1	–2.3
Other**^†††^**	1,053 (0.3) [0.3–.03]	1,085 (0.3) [0.3–0.3]	0	0
**Female**				
**Total**	**10,583 (6.2) [6.1–6.3]**	**10,255 (6.0) [5.9–6.1]**	–0.2**	–3.2**
**Race/Ethnicity^††^**				
American Indian/Alaska Native	136 (11.1) [9.2–13.0]	145 (12.1) [10.1–14.1]	1.0	9.0
Asian	394 (3.8) [3.4–4.2]	392 (3.7) [3.3–4.0]	–0.1	–2.6
Black or African American	616 (2.9) [2.6–3.1]	624 (2.9) [2.7–3.2]	0	0
Native Hawaiian/Other Pacific Islander	12 (—)	18 (—)	—	—
White	8,418 (8.0) [7.8–8.2]	8,046 (7.7) [7.5–7.9]	–0.3**	–3.8**
Multiracial	132 (4.5) [3.7–5.3]	122 (3.9) [3.2–4.7]	–0.6	–13.3
Hispanic	844 (2.9) [2.7–3.0]	886 (3.0) [2.8–3.2]	0.1	3.4
Unknown	31 (—)	22 (—)	—	—
**Age group, yrs^§§^**				
10–14	206 (2.0) [1.7–2.3]	203 (2.0) [1.7–2.3]	0	0
15–24	1,222 (5.8) [5.5–6.2]	1,154 (5.5) [5.2–5.9]	–0.3	–5.2
25–34	1,670 (7.4) [7.1–7.8]	1,526 (6.8) [6.4–7.1]	–0.6**	–8.1**
35–44	1,742 (8.4) [8.0–8.8]	1,710 (8.2) [7.8–8.6]	–0.2	–2.4
45–54	2,143 (10.2) [9.7–10.6]	2,156 (10.4) [10.0–10.9]	0.2	2.0
55–64	2,069 (9.5) [9.1–9.9]	1,948 (8.9) [8.5–9.3]	–0.6**	–6.3**
65–74	1,011 (6.2) [5.8–6.6]	985 (5.9) [5.5–6.2]	–0.3	–4.8
75–84	364 (4.2) [3.8–4.6]	410 (4.6) [4.1–5.0]	0.4	9.5
≥85	151 (3.6) [3.0–4.2]	158 (3.7) [3.2–4.3]	0.1	2.8
**Urbanization^¶¶^**				
Large central metropolitan	2,701 (5.1) [4.9–5.3]	2,682 (5.0) [4.8–5.2]	–0.1	–2.0
Large fringe metropolitan	2,555 (5.9) [5.6–6.1]	2,457 (5.6) [5.4–5.9]	–0.3	–5.1
Medium metropolitan	2,428 (6.8) [6.5–7.1]	2,400 (6.7) [6.4–7.0]	–0.1	–1.5
Small metropolitan	1,094 (7.3) [6.8–7.7]	1,106 (7.3) [6.9–7.8]	0	0
Micropolitan (nonmetropolitan)	1,070 (7.9) [7.4–8.4]	918 (6.9) [6.4–7.3]	–1.1**	–12.7**
Noncore (nonmetropolitan)	735 (8.2) [7.6–8.8]	692 (7.9) [7.3–8.5]	–0.3	–3.7
**Mechanism of injury**				
Cut/Pierce	162 (0.1) [0.1–0.1]	152 (0.1) [0.1–0.1]	0	0
Drowning	195 (0.1) [0.1–0.1]	187 (0.1) [0.1–0.1]	0	0
Fall	308 (0.2) [0.2–0.2]	333 (0.2) [0.2–0.2]	0**	0**
Fire/Flame	61 (0) [0–0.1]	59 (0) [0–0.1]	0	0
Firearm	3,331 (1.9) [1.8–2.0]	3,216 (1.9) [1.8–1.9]	0	0
Poisoning	3,100 (1.7) [1.7–1.8]	3,079 (1.7) [1.7–1.8]	0	0
Suffocation	3,163 (2.0) [1.9–2.0]	2,971 (1.8) [1.8–1.9]	–0.2**	–10.0**
Other**^†††^**	263 (0.2) [0.1–0.2]	258 (0.2) [0.1–0.2]	0	0
**Male**				
**Total**	**37,761 (22.8) [22.6–23.0]**	**37,256 (22.4) [22.1–22.6]**	–0.4**	–1.8**
**Race/Ethnicity^††^**				
American Indian/Alaska Native	409 (33.6) [30.3–36.9]	401 (33.0) [29.7–36.3]	–0.6	–1.8
Asian	921 (10.0) [9.3–10.6]	950 (10.1) [9.4–10.7]	0.1	1.0
Black or African American	2,406 (12.2) [11.7–12.7]	2,491 (12.5) [12.0–13.0]	0.3	2.5
Native Hawaiian/Other Pacific Islander	61 (19.8) [15.0–25.6]	72 (22.1) [17.3–28.0]	2.3	11.6
White	29,997 (28.6) [28.3–29.0]	29,382 (28.1) [27.7–28.4]	–0.5**	–1.7**
Multiracial	382 (13.8) [12.3–15.3]	405 (14.2) [12.7–15.7]	0.4	2.9
Hispanic	3,469 (12.1) [11.7–12.5]	3,445 (11.6) [11.2–12.0]	–0.5	–4.1
Unknown	116 (—)	110 (—)	—	—
**Age group, yrs^§§^**				
10–14	390 (3.7) [3.3–4.0]	331 (3.1) [2.8–3.5]	–0.6**	–16.2**
15–24	4,989 (22.7) [22.1–23.3]	4,800 (22.0) [21.4–22.6]	–0.7	–3.1
25–34	6,350 (27.4) [26.7–28.0]	6,533 (28.0) [27.3–28.7]	0.6	2.2
35–44	5,779 (28.1) [27.4–28.8]	5,815 (28.0) [27.3–28.7]	–0.1	–0.4
45–54	6,202 (30.2) [29.4–30.9]	5,856 (29.0) [28.3–29.8]	–1.2**	–4.0**
55–64	6,471 (31.7) [31.0–32.5]	6,290 (30.7) [29.9–31.4]	–1.0	–3.2
65–74	3,963 (27.8) [27.0–28.7]	3,882 (26.4) [25.6–27.2]	–1.4**	–5.0**
75–84	2,516 (37.4) [35.9–38.8]	2,567 (36.7) [35.3–38.1]	–0.7	–1.9
≥85	1,097 (47.2) [44.4–50.0]	1,171 (49.3) [46.5–52.1]	2.1	4.4
**Urbanization^¶¶^**				
Large central metropolitan	9,277 (18.3) [18.0–18.7]	9,080 (17.8) [17.5–18.2]	–0.5	–2.7
Large fringe metropolitan	8,473 (20.5) [20.1–21.0]	8,383 (20.0) [19.6–20.5]	–0.5	–2.4
Medium metropolitan	8,434 (24.5) [24.0–25.0]	8,389 (24.3) [23.7–24.8]	–0.2	–0.8
Small metropolitan	4,279 (28.3) [27.4–29.1]	4,221 (27.9) [27.0–28.7]	–0.4	–1.4
Micropolitan (nonmetropolitan)	4,267 (30.6) [29.6–31.5]	4,091 (29.5) [28.6–30.4]	–1.1	–3.6
Noncore (nonmetropolitan)	3,031 (31.0) [29.8–32.1]	3,092 (32.1) [31.0–33.3]	1.1	3.5
**Mechanism of injury**				
Cut/Pierce	735 (0.4) [0.4–0.4]	769 (0.4) [0.4–0.5]	0	0
Drowning	327 (0.2) [0.2–0.2]	319 (0.2) [0.2–0.2]	0	0
Fall	841 (0.5) [0.5–0.5]	850 (0.5) [0.5–0.5]	0	0
Fire/Flame	153 (0.1) [0.1–0.1]	128 (0.1) [0.1–0.1]	0	0
Firearm	21,101 (12.6) [12.4–12.7]	20,725 (12.3) [12.1–12.4]	–0.3**	–2.4**
Poisoning	3,137 (1.9) [1.8–1.9]	3,046 (1.8) [1.7–1.9]	–0.1	–5.3
Suffocation	10,677 (6.7) [6.5–6.8]	10,592 (6.6) [6.5–6.7]	–0.1	–1.5
Other**^†††^**	790 (0.5) [0.4–0.5]	827 (0.5) [0.5–0.5]	0	0

**Abbreviation:** CI = confidence interval.

* Age-adjusted death rates (per 100,000) were calculated by using the direct method and the 2000 U.S. standard population. Rates and CIs are rounded to one digit and as a result might not exactly match similar rates published elsewhere. Suicides for persons aged <10 years were included in the total numbers and age-adjusted rates but are not shown as part of age groups because determining suicidal intent in younger children can be difficult, and case counts were <20, indicating unstable rates.

^†^ Suicide deaths were identified by using *International Classification of Diseases, Tenth Revision* underlying cause-of-death codes U03, X60–X84, and Y87.0.

**^§^** The rate in 2019 minus the rate in 2018.

^¶^ The (2019 rate minus 2018 rate) divided by 2018 rate multiplied by 100.

** P≤0.05 for difference between 2018 and 2019. Z-tests were used if the number of deaths was ≥100 in both 2018 and 2019; nonoverlapping confidence intervals based on the gamma method were used if the number of deaths was <100 in 2018 or 2019.

^††^ Data for Hispanic origin should be interpreted with caution; studies comparing Hispanic origin on death certificates and on Census surveys have shown inconsistent reporting on Hispanic ethnicity. Potential racial misclassification might lead to underestimates for certain categories, primarily non-Hispanic American Indian/Alaska Native and non-Hispanic Asian/Pacific Islander decedents. Single-race estimates are presented and might not be comparable to earlier years produced by bridging multiple races to a single race choice. Hispanic and unknown ethnicity include persons of any race. Racial groups exclude persons of Hispanic or unknown ethnicity.

**^§§^** Crude rates per 100,000 are presented for age groups.

^¶¶^ Urbanization level of the decedent’s county of residence was categorized by using the 2013 National Center for Health Statistics Urban–Rural Classification Scheme for Counties (https://www.cdc.gov/nchs/data_access/urban_rural.htm). The classification levels for counties are as follows: 1) large central metropolitan: part of a metropolitan statistical area with ≥1 million population and covers a principal city; 2) large fringe metropolitan: part of a metropolitan statistical area with ≥1 million population but does not cover a principal city; 3) medium metropolitan: part of a metropolitan statistical area with ≥250,000 but <1 million population; 4) small metropolitan: part of a metropolitan statistical area with <250,000 population; 5) micropolitan: part of a micropolitan statistical area (has an urban cluster of ≥10,000 but <50,000 population); and 6) noncore (nonmetropolitan): not part of a metropolitan or micropolitan statistical area.

*** Because of the change in the composition of the population during 2018–2019, the rounded age-adjusted rate increased 100%, from 0.1 to 0.2, as the number of drowning deaths decreased from 522 to 506. Confidence intervals are the same for both age-adjusted rate estimates.

**^†††^** “Other” mechanisms of injury include other land transport, struck by/against, other specified, and unspecified.

Rates in 2019 were highest among persons aged ≥85 years (20.1 per 100,000), with counts highest among persons aged 55–64 years (8,238) (Table). The number of suicides among males was highest for those aged 25–34 years, a change from 2018, when counts were highest among males aged 55–64 years. Among females, the largest counts and highest rate of suicide were among those aged 45–54 years. Rates declined significantly among persons aged 15–24 years (3.4%; 14.5 to 14.0), 55–64 years (4.0%; 20.2 to 19.4), and 65–74 years (4.9%; 16.3 to 15.5). Significant declines also occurred among males aged 10–14 years (16.2%; 3.7 to 3.1), females aged 25–34-years (8.1%; 7.4 to 6.8), males aged 45–54 years (4.0%; 30.2 to 29.0), females aged 55–64 years (6.3%; 9.5 to 8.9), and males aged 65–74 years (5.0%; 27.8 to 26.4).

Suicide rates in 2019 were lowest in large central metropolitan areas (11.2 per 100,000) and increased as the level of urbanization declined, with noncore (nonmetropolitan) areas having the highest rate (20.1 per 100,000); this stepped pattern occurred among both females and males. Rates declined from 2018 to 2019 in two county urbanization levels: large fringe metropolitan (3.1%) and micropolitan (nonmetropolitan) (5.7%). Rates also declined among females in micropolitan (nonmetropolitan) areas.

In 2019, the largest proportion of suicides occurred by use of firearms (50.4%), with a rate of 6.8 per 100,000. Whereas males were most likely to die from a firearm-related injury (55.6%) females were equally likely to die from firearm use (31.4%), poisoning (30.0%), and suffocation (e.g., hanging) (29.0%). The rate of firearm suicides declined significantly from 2018 to 2019, by 2.9% (from 7.0 to 6.8 per 100,000), overall, likely driven by a 2.4% decline in their use among males (from 12.6 to 12.3 per 100,000); the rate of firearm suicide among females did not change. The rate of suicide by suffocation among females decreased significantly (10.0%; from 2.0 to 1.8 per 100,000). Rates of suicide by all other mechanisms did not change significantly overall or among females or males.

Firearms were the most common mechanism of suicide in 2019 in all county urbanization levels (Figure 1). The percentage of suicides by firearm in 2019 increased in a stepped pattern from the most urban counties (41.7%) to the most rural (i.e., least urban) counties (62.5%). Conversely, suffocation, the second most prevalent mechanism of suicide, followed a largely stepped decrease from most urban (31.4%) to least urban (28.5%) counties; suicides by poisoning followed a similar pattern, from 14.5% (most urban) to 12.9% (least urban).

**FIGURE 1:**
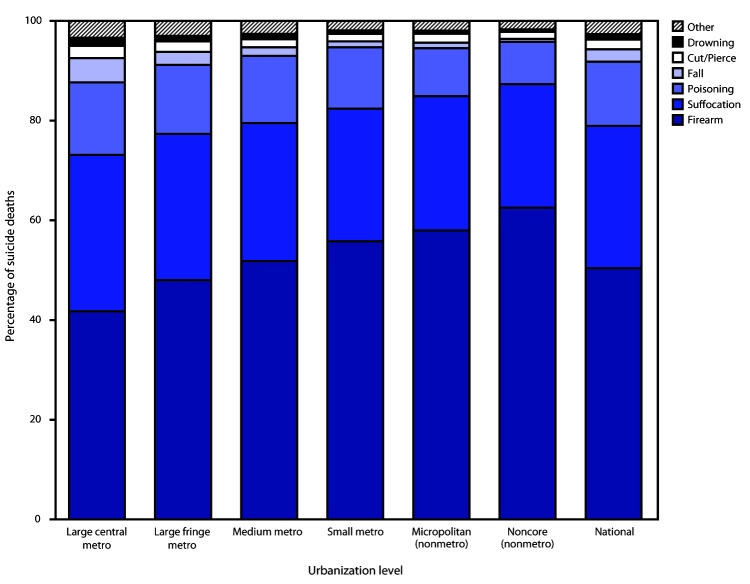
Suicide* mechanism of injury,^†^ by level of urbanization^§^ — National Vital Statistics System, United States, 2019 * Suicide deaths were identified by using *International Classification of Diseases, Tenth Revision* underlying cause-of-death codes U03, X60–X84, and Y87.0. ^†^ “Other” mechanisms of injury include other land transport, struck by/against, other specified, and unspecified. ^§^ Urbanization level of the decedent’s county of residence was categorized by using the 2013 National Center for Health Statistics Urban–Rural Classification Scheme for Counties (https://www.cdc.gov/nchs/data_access/urban_rural.htm). The classification levels for counties are as follows: 1) large central metropolitan (large central metro): part of a metropolitan statistical area with ≥1 million population and covers a principal city; 2) large fringe metropolitan (large fringe metro): part of a metropolitan statistical area with ≥1 million population but does not cover a principal city; 3) medium metropolitan (medium metro): part of a metropolitan statistical area with ≥250,000 but <1 million population; 4) small metropolitan (small metro): part of a metropolitan statistical area with <250,000 population; 5) micropolitan (nonmetro): part of a micropolitan statistical area (has an urban cluster of ≥10,000 but <50,000 population); and 6) noncore (nonmetro): not part of a metropolitan or micropolitan statistical area.

The overall suicide rate declined significantly from 2018 to 2019 in five states (Idaho, Indiana, Massachusetts, North Carolina, and Virginia) (Figure 2). The suicide rate among females declined significantly in three states (Indiana, Missouri, and Washington), and rates among males declined significantly in five states (Florida, Kentucky, Massachusetts, North Carolina, and West Virginia) (Supplementary Table, https://stacks.cdc.gov/view/cdc/102794). The largest significant overall decline occurred in Idaho (14.6%). Among females, the largest significant decline occurred in Indiana (29.7%). Among males, the largest significant decline occurred in West Virginia (16.1%). The suicide rate increased significantly overall in Hawaii (30.3%) and Nebraska (20.1%), among females in Minnesota (39.6%), and among males in Hawaii (35.1%) and Wyoming (39.6%).

**FIGURE 2:**
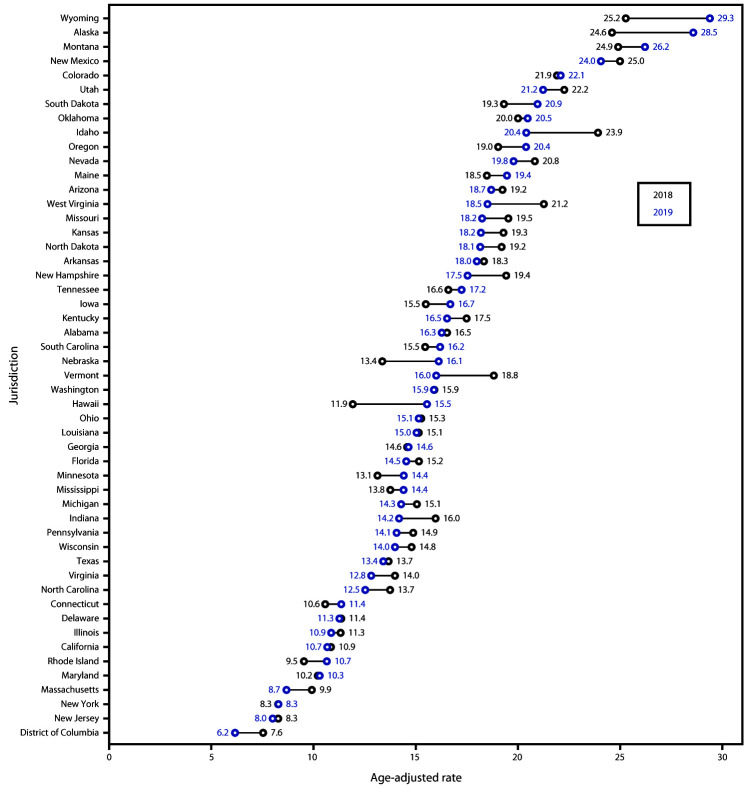
Overall age-adjusted rate*^,†^ of suicide,^§^ by state — National Vital Statistics System, United States, 2018 and 2019 * Age-adjusted death rates per 100,000 population were calculated by using the direct method and the 2000 U.S. standard population. Rates are rounded to one digit. ^†^ States with statistically significant changes (p≤0.05); Z-tests were used if the number of deaths was ≥100 in both 2018 and 2019; nonoverlapping confidence intervals based on the gamma method were used if the number of deaths was <100 in 2018 or 2019. States with statistically significant changes were Hawaii, Idaho, Indiana, Massachusetts, Nebraska, North Carolina, and Virginia. ^§^ Suicide deaths were identified by using *International Classification of Diseases, Tenth Revision* underlying cause-of-death codes U03, X60–X84, and Y87.0.

## Discussion

The declines in suicide rates in 2019 are encouraging after 13 consecutive years of rate increases (*1*). From 2018 to 2019, the suicide rate decreased by 2.1%, with significant declines among both females and males and among multiple age groups. Suicide rates declined in large fringe metropolitan areas and micropolitan areas and in five states, overall. Particularly encouraging was the significant decline in firearm suicides, the mechanism of suicide that is most common and most lethal (*6*). However, few significant declines were observed by race/ethnicity, most states did not experience significant changes, and a small number of states experienced increased rates, underscoring persisting disparities in 2019.

Research has shown that suicide is preventable and that risks for suicide extend beyond mental health and lack of access to mental health treatment alone (*7*). Suicide prevention must focus on the constellation of associated factors, including mental illness, substance misuse, high conflict or violent relationships, social isolation, job and financial problems, lack of community connectedness, barriers to suicide-related care, and access to lethal means among persons at risk (*7*).

As the United States continues to respond to the coronavirus disease 2019 (COVID-19) pandemic and its long-term impacts on isolation, stress, economic insecurity, and worsening mental health and wellness, prevention is more important than ever. Past research indicates that suicide rates remain stable or decline during infrastructure disruption (e.g., natural disasters), only to rise afterwards as the longer-term sequalae unfold in persons, families, and communities (*8*).

A comprehensive approach to suicide prevention is urgently needed in all states to continue the initial progress made in 2019. A comprehensive approach is one that relies on use of data to drive decision-making and robust implementation and evaluation of prevention strategies with the best available evidence that address the range of factors associated with suicide, especially among populations disproportionately affected (https://www.cdc.gov/injury/fundedprograms/comprehensive-suicide-prevention/index.html). Such strategies are all the more relevant in the midst of the COVID-19 pandemic and include those focused on strengthening economic supports, expanding access to and delivery of care (e.g., telehealth), promoting social connectedness, creating protective environments including reducing access to lethal means among persons at risk, teaching coping and problem-solving skills, identifying and supporting persons at risk, and lessening harms and preventing future risk (e.g., safe media reporting on suicide) (*3*).

The findings in this report are subject to at least two limitations. First, caution must be used when interpreting rate decreases from 1 year to the next because rates might be unstable, especially in smaller segments of the population, and declines observed in a single year cannot be interpreted as a trend. Second, evidence over several decades suggests that suicides are undercounted on death certificates for various reasons, including the higher burden of proof to classify a death as a suicide (versus proof needed to classify other manners of death), stigma, and lack of autopsies or thorough investigations (*9*); thus, suicide rates might be underestimated in 2018 and 2019.

Suicide is preventable, and effective approaches to both reduce suicide risk factors and increase protective factors are available. Comprehensive prevention efforts are critical to realize further declines in suicide and to reach the national goal to reduce suicide rates by 20% by 2025 (*10*). Resources are available that states and communities can use to better understand suicide, prioritize evidence-based comprehensive suicide prevention, and save lives (*3*).

**Summary**

**What is already known about this topic?**

Suicide is preventable. In 2019, approximately 47,500 lives were attributed to suicide. From 2018 to 2019, the suicide rate declined for the first time in more than a decade.

**What is added by this report?**

Suicide rates declined overall by 2.1%, among females by 3.2%, and among males by 1.8%, as well as in five states, certain demographic groups, and by certain mechanisms of suicide; however, disparities persist.

**What are the implications for public health practice?**

To build on 2019 progress, CDC’s Preventing Suicide: A Technical Package of Policy, Programs, and Practices supports a comprehensive approach to prevention. Implementing such an approach, especially in disproportionately affected populations (e.g., American Indian/Alaska Natives), is needed in all states.
